# Reliable biomarkers for diabetic nephropathy using machine learning-assisted contrast-enhanced ultrasonography and clinical characteristics

**DOI:** 10.1007/s10238-025-01837-2

**Published:** 2025-10-31

**Authors:** Lin Lin, Lin Yan, Nan Li, Yiru Wang, Yukun Luo

**Affiliations:** 1 Department of Ultrasound, The First Medical Center, Chinese PLA General Hospital, 28 Fuxing Rd, Beijing, 100853 China; 2 PLA Medical College, Beijing, China

**Keywords:** Contrast-enhanced ultrasound, Clinical characteristics, Diabetic nephropathy, Biomarker

## Abstract

**Objective:**

To utilize machine learning techniques to screen contrast-enhanced ultrasound (CEUS) parameters and clinical characteristics, aiming to differentiate diabetic nephropathy (DN) from non-diabetic renal disease (NDRD) in patients with diabetic kidney injury.

**Methods:**

Data from 120 diabetic patients (240 kidneys) with chronic kidney disease (CKD) were analyzed. The data included basic clinical features for each kidney and renal vascular data obtained through CEUS. Statistical analysis, tenfold cross-validation and random forest method were used for data processing. Receiver operating characteristic (ROC) curves were employed to depict the diagnostic performance of the indicators.

**Results:**

The random forest model integrating CEUS parameters and clinical characteristics achieved an average classification accuracy of 87.6% in differentiating kidney injury types. ROC curve analysis showed an AUC of 0.918.

**Conclusion:**

Through machine learning, CEUS quantitative parameters and clinical features of the screened model can be used as important noninvasive biomarkers to identify kidney injury in T2DM patients with DN. Ai-assisted CEUS and specific clinical features are a fast and reliable tool for DN screening.

## Introduction

Chronic kidney disease (CKD) defined as proteinuria (urinary albumin to creatinine ratio [UACR] ≥ 30 mg /g/d) or glomerular filtration rate (GFR) less than 60 ml /min/1.73 m2 for at least 3 months [1]. By 2027, the global incidence of CKD is expected to rise to 436.60 million cases, an increase of 5.8% from 2022, and most CKD cases (about 80%) are still undiagnosed [2]. Moreover, the proportion of diabetic nephropathy (DN) in CKD is constantly increasing, and it is an important cause of end-stage renal disease, bringing a heavy disease burden to patients [3]. As a common microvascular complication in diabetic patients, diabetic nephropathy progresses rapidly and has a poor prognosis [4]. Its pathological mechanisms include the formation of advanced glycosylation end products (AGEs), activation of polyol pathway, release of inflammatory factors, increase of glomerular hemodynamic pressure, expansion of mesangial stroma and interstitial fibrosis, which gradually lead to glomerular sclerosis, especially the Kimmelstiel-Wilson nodule formed by severe hyperplasia of glomerular mesangial stroma [5–7]. These lesions cause changes in the microvessels of patients with diabetic nephropathy, leading to various cellular dysfunctions of the kidneys, ultimately leading to progressive renal failure [8]. Evaluation and monitoring of renal blood perfusion are of great significance for the auxiliary diagnosis and detection of diabetic nephropathy. The application of ultrasonomics for the noninvasive, real-time assessment of kidney parameters, in conjunction with artificial intelligence (AI) for the advanced analysis of both 2D ultrasound and contrast-enhanced ultrasound data, holds significant promise for the auxiliary diagnosis, detection and evaluation of diabetic nephropathy (DN).

In addition, clinically, the differential diagnosis of DN and non-diabetic nephropathy (NDRD) in diabetic patients is very important due to the completely different treatment and prognosis [4]. Therefore, whether a reliable biomarker can be found to identify DN is of great significance for patients with CKD, especially those with diabetic kidney injury.

## Materials and methods

### Patients

**Figure 1:**
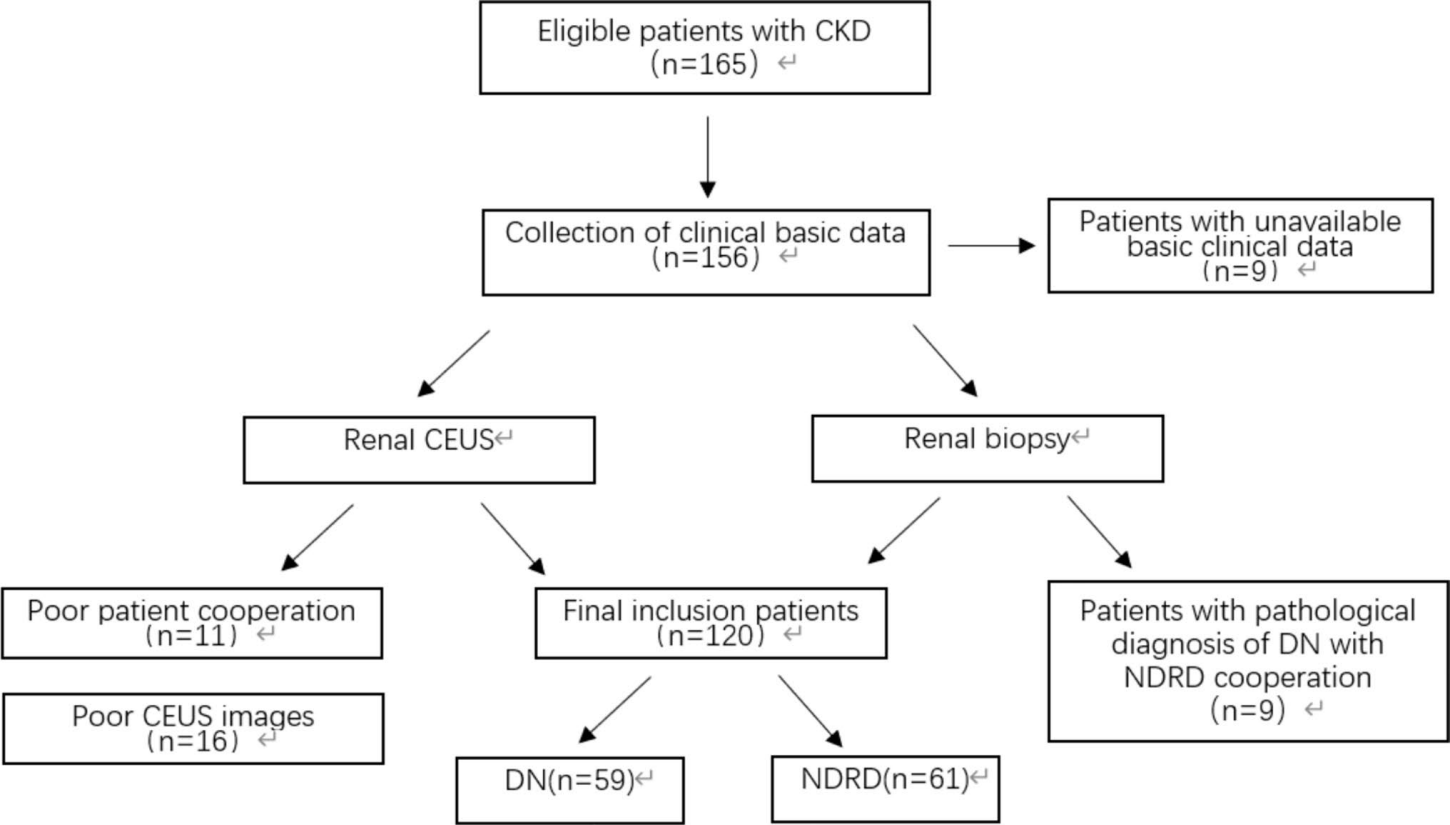
Enrollment process.

The study included a total of 120 patients with type 2 diabetes with CKD who were treated at the Chinese PLA General Hospital between May 2017 and January 2020. This study has been approved by the hospital ethics committee (approval number S2017-152-02). All participants signed a CEUS informed consent form prior to the examination. The basic clinical characteristics of all patients were recorded (Table 1). According to pathological results, 120 subjects were divided into two groups: DN group and NDRD group (Figure 1).

**Table 1:** Baseline characteristics

Variable	DN	NDRD	*χ*^*2*^/* t* Test
*N* (subjects/kidneys)	59/118	61/122	
Gender (male/female)	43/16	39/22	*χ*^*2*^=1.109
Age (years)	51.71±10.65	48.15±13.64	*t*=2.536
BMI (kg/m^2^)	26.73±3.60	26.25±3.82	*t*=0.497
Serum Creatinine(umol/L)	162.77±83.71	108.49±58.48	*t*=5.840
eGFR–EPI (mL/ (min·1.73 m^2^))	42.87 ± 24.77	67.96 ± 31.63	*t*=-6.832
Blood glucose(mmol/L)	5.45±1.95	5.43±1.40	*t*=0.122
HbA1c	0.07±0.01	0.06±0.01	*t*=5.109
Urine protein concentration (g/L)	1.23±1.04	0.92±0.99	*t*=2.318

The study population consisted of patients scheduled for diagnostic renal puncture. All the enrolled patients fulfilled these inclusion criteria: (1) adult patients older than 18 years of age; (2) patients with CKD; (3) clinical diagnosis of type 2 diabetes mellitus; (4) a definite pathological diagnosis of renal puncture; (5) the patient underwent CEUS examination before the renal puncture; exclusion criteria were: (1) pathological diagnosis of DN with NDRD; (2) the basic clinical characteristics were incomplete; (3) CEUS image quality was substandard, or image analysis consistency was poor; (4) hypersensitivity to ultrasound contrast agent (UCA) or contraindications to contrast agent instructions; (5) patients with malignant nodules, huge cysts, serious cardiovascular and cerebrovascular diseases, serious lung diseases.

### CEUS assessment

In order to rule out the effect of blood pressure on renal blood vessels, the blood pressure of the subjects should be controlled below 140/90mmHg and above 100/60 mmHg before CEUS examination. An ultrasound instrument (Siemens, S2000, Germany) with a probe-selective convex multi-frequency transducer (4–6 MHz) was used to perform CEUS on the patient. Both kidneys of each patient were examined by an experienced physician with more than 10 years of experience in US and CEUS. The ultrasonic contrast agent was selected with sulfur hexafluoride (SonoVue, Braco, Italy) at a dose of 0.5 ml and dissolved in 5 mL 0.9% sodium chloride solution. The kidney signal changes were observed for 180 seconds. Meanwhile, the images were saved.

The software analysis of the saved CEUS images existed a certain degree of subjectivity because the region of interest (ROI) was manually selected in the analysis process. Therefore, image analysis was conducted separately by two experienced physicians and consistency analysis was performed.

### Data acquisition and machine learning

**Figure 2:**
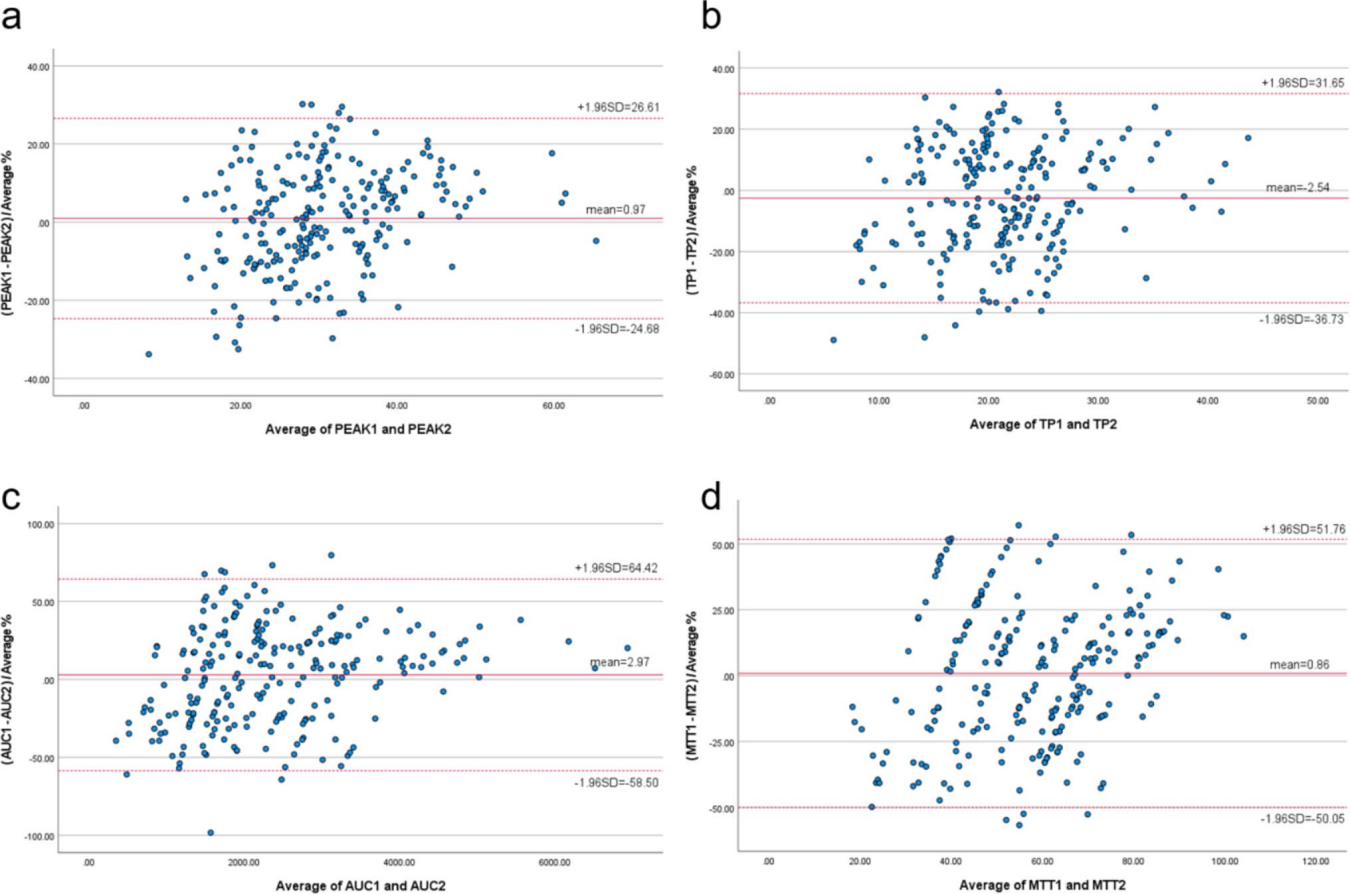
Bland–Altman plots of the differences between the right contrast-enhanced ultrasound parameters obtained by physician 1 and 2 (y- axis) and the mean of the parameters (x- axis). **a**. PEAK; **b**. TP; **c**. AUC; **d**. MTT. The red solid line indicates the mean value of all measurements (n = 40), and 95% LOA (mean ± 1.96 SDs). AUC, area under the curve; CEUS, contrast-enhanced ultrasound; LOA, limits-of-agreement; MTT, mean transit time; PEAK, peak intensity; TP, parenchyma time.

Four parameters were obtained through the built-in quantitative analysis software of CEUS. Peak intensity (PEAK; defined as the maximum signal strength achieved by contrast agent enhancement in the region of interest during CEUS), time-to-peak (TP; defined as the time required from the beginning of the injection of contrast agent to the peak intensity of contrast enhancement within the area of interest), area under the curve (AUC; defined as the area under the CEUS time–intensity curve). Mean transit time (MTT; defined as the average residence time of contrast agent microbubbles in the region of interest). Cases with poor image quality were excluded, and a consistent analysis of the CEUS results obtained by two experienced physicians (Table 2) was also performed, which showed good consistency (Table 3 and Figure 2). The final CEUS parameter result was determined by using the mean.

**Table 2:** CEUS parameters obtained by physician 1 and 2

	Physician 1		Physician 2	
	DN	NDRD	DN	NDRD
PEAK (%)	27.45±8.62	25.95±7.05	27.28±7.74	26.31±5.99
TP (s)	22.87±7.59	20.80±6.80	22.90±6.78	21.62±6.61
AUC (%s)	2359.21±1310.87	1960.88±919.02	2289.34±1154.96	1933.45±786.90
MTT (s)	61.12±21.35	55.33±16.28	59.61±15.80	57.13±16.60

**Table 3:** The ICCs, 95% CIs and Bland–Altman plot analysis obtained by physician 1 and 2

	ICC	95% CIs	Mean ± SD (%)	95% LOA (%)
PEAK	0.956	0.943–0.966	0.97±13.08	−24.68~26.61
TP	0.917	0.893–0.936	−2.54±17.45	−36.73~31.65
AUC	0.897	0.867–0.920	2.97±31.36	−58.50~ 64.43
MTT	0.824	0.773–0.863	0.86±25.97	−50.05~51.76

The machine learning method was adopted to standardize CEUS and clinical basic data, and the selection of the first 8 variables was based on the results of a single-factor logistic regression analysis, which evaluated the association between each individual predictor and the binary outcome (DN/NDRD). Regarding tenfold cross-validation, it is mainly used to evaluate the stability and generalization ability of the final model. And then the data were randomly divided into 10 groups with the same number of each group to form a training set and a test set. The training set was divided into 10 groups for cross-validation, each containing 90% of the training data and 10% of the validation data. The training set data are then fitted into a random forest model, which is used to classify the data from the test set [9, 10]. Based on the trained model, DN was identified in CKD patients and the classification accuracy was calculated.

The statistical analyses were conducted using SPSS v27.0 and R language (version 4.4.2). Continuous variables were presented as mean ± standard deviation, and differences between groups were assessed using the independent samples t-test; categorical variables were summarized as counts (percentages), and the χ^2^ test was applied to evaluate differences in distribution between groups. A two-tailed p-value < 0.05 was considered statistically significant for all tests. Consistency analysis was evaluated using the intraclass correlation coefficient (ICC), and a B-A diagram was generated to visually assess the consistency. Univariate logistic regression was performed in R (version 4.4.2) to identify the most significant indicators associated with the outcome, with significance determined using the Wald test (p < 0.05 as the cutoff).

In addition, SPSS v27.0 software was used for data description statistics, consistency analysis, single-factor analysis, etc., and B-A chart was drawn.

## Results

Data from 120 CKD patients with type 2 diabetes (240 kidneys) were included in this study, including 82 men and 38 women. All patients underwent renal puncture after CEUS and obtained renal pathology results. All renal biopsy procedures and pathological assessments were conducted in strict accordance with standardized protocols, with diagnoses of diabetic nephropathy (DN) and non-diabetic renal disease (NDRD) rigorously validated against the Renal Pathology Society (RPS) 2010 classification system, thereby ensuring the highest standards of precision and rigor in characterizing the pathological features of each case [11, 12].

**Figure 3:**
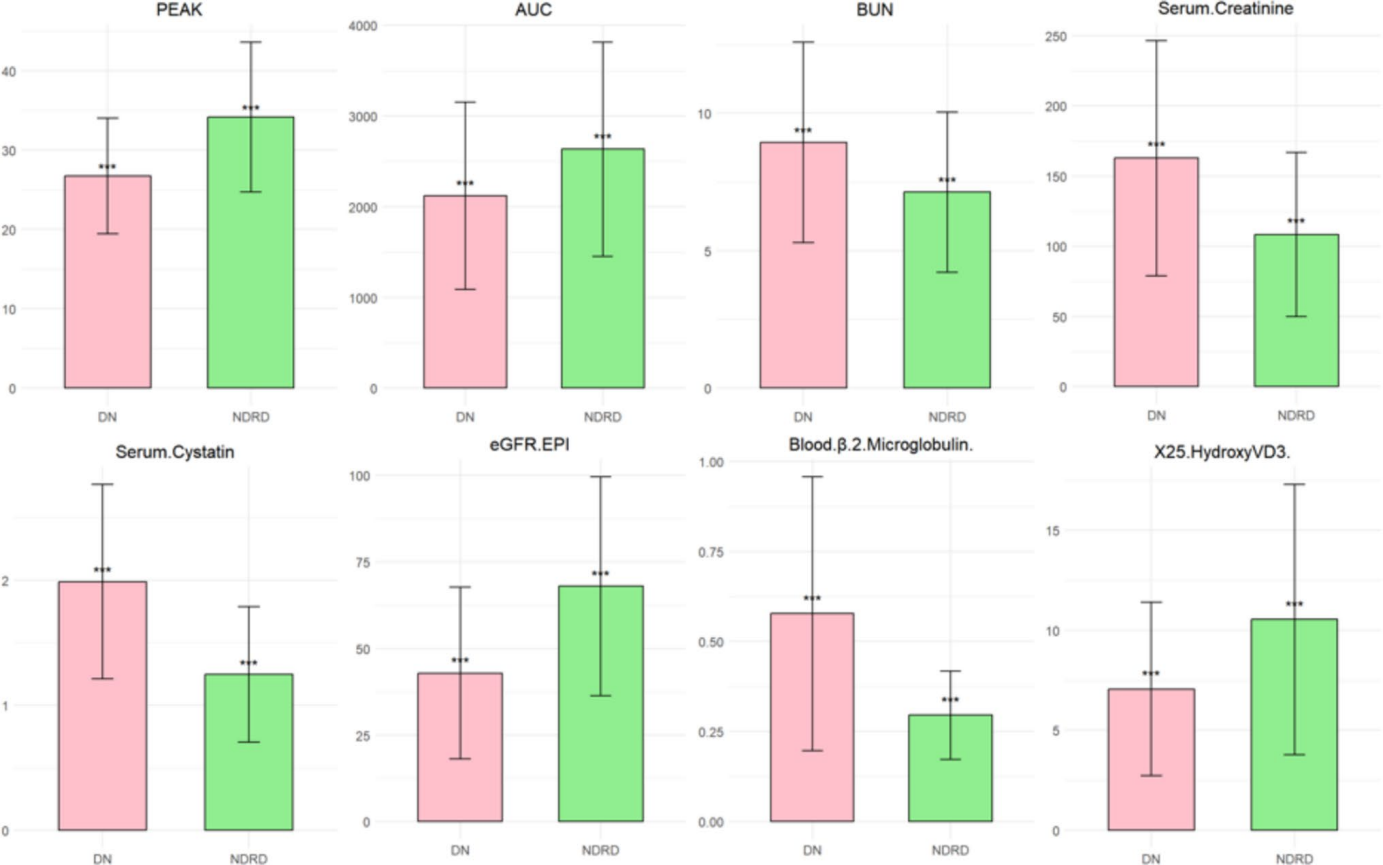
Using the R programming language, eight most significant variables were selected.

A machine learning approach was used to standardize the data, assess the importance of explanatory variables through tenold cross-validation and ultimately identified the eight most relevant variables out of 21 (Figure 3). Z-score standardization was applied to the dataset to ensure comparability across features when evaluating their importance. Z-score standardization preserves the original distribution of each feature, merely shifting and scaling it. This was particularly important given the non-uniform distribution of some variables, as it allowed us to retain the inherent variability while enabling fair comparisons of feature importance. The data were then further processed using tenfold cross-validation and random forest models to screen DN and NDRD. As shown in Table 4, the classification accuracy of the tenfold cross-validation method and the average classification accuracy of the random forest method were presented. In the 240 kidneys with CKD, the average classification accuracy was calculated to be 87.6%. Other indicators for evaluating the performance of the comprehensive assessment classification model are listed in Table 5.

**Table 4:** Classification accuracy of DN patients from the T2DM patients based on random forest method (tenfold cross-validation).

Random Forest	T2DM patients (%)
FOLD01	84.4
FOLD02	94.1
FOLD03	88.2
FOLD04	88.2
FOLD05	94.4
FOLD06	88.2
FOLD07	84.4
FOLD08	90.6
FOLD09	82.4
FOLD10	80.6
Average	87.6

**Table 5:** The comprehensive evaluation indicators of this classification model

Performance Metrics	Value
Kappa Coefficient	0.718
Sensitivity	0.829
Specificity	0.889
Positive Predictive Value	0.879
Negative Predictive Value	0.842
ROC	0.918

## Discussion

Diabetes is a complex metabolic disease, which can lead to a variety of complications, chronic damage to a variety of organs and tissues and a serious threat to the quality of life of patients [13]. In recent years, the incidence of diabetic nephropathy has increased year by year and has become the main source of end-stage nephropathy [14]. However, in diabetic patients with kidney injury, the diagnosis of diabetic nephropathy depends on the pathological results of kidney puncture, but it can cause damage to the blood vessels around the calyces, and even the risk of perforation of the abdominal organs or pleura [15, 16]. Therefore, for diabetic patients with chronic kidney injury, it is very important to find a better noninvasive method to identify diabetic nephropathy for targeted treatment.

The types of nephropathy that induce kidney damage in diabetic patients are diverse and intricate. Besides diabetic nephropathy, conditions such as chronic renal tubular injury, glomerulonephritis, IgA nephropathy and hypertensive nephropathy can also occur. Moreover, two or more types of nephropathy may coexist. The accuracy of diagnosing different types of kidney diseases significantly influences their clinical treatment strategies and goals, thereby altering the course and prognosis of kidney diseases. Identifying potential non-diabetic renal diseases (NDRDs) holds great clinical significance [17].

Whether biochemical indicators and ultrasonic medical indicators can be organically combined to evaluate the kidney status of patients more comprehensively, artificial intelligence came into being. Through the random forest algorithm of machine learning, the laboratory inspection and CEUS parameters are incorporated into the random forest model, and the data are automatically learned, the rules are found, and the rules are used to make predictions and decisions [18].

CEUS can quantitatively evaluate renal microcirculation perfusion through intravenous injection of microbubble contrast agent combined with real-time dynamic imaging technology, and its quantitative parameters can noninvasive and sensitive mirror the hemodynamic changes caused by microvascular injury in the glomeruli and renal tubulointerstitium [19]. This provides valuable insights into the microvascular status of the kidneys, enabling early detection and monitoring of potential renal pathologies related to microvascular injury. In this study, among the top eight important factors screened by single factors, there are two CEUS parameters, namely PEAK and AUC. Y. Wang [20] et al. also reached a similar conclusion, suggesting that quantification of CEUS parameters is a feasible parameter to identify DN, PE and AUC in patients with diabetes complicated with kidney injury.

In this model, in addition to eGFR/EPI, serum creatinine and serum protein concentrations [21–23], which are routinely used to assess renal function, biochemical indicators were also included in β2 microglobulin, serum cystatin and total cholesterol.

β2 microglobulin is a low molecular weight endogenous protein secreted mainly by lymphocytes and other nucleated cells, and is a sensitive indicator of glomerular filtration function and a marker of renal tubule injury [24]. Takayuki Uemura [25] et al. suggested that serum β2-MG could be predictive of kidney failure by studying patients with biopsy-confirmed diabetic nephropathy.

Serum cystatin C is produced stably by all nucleated cells and is completely filtered through the glomeruli. It is a sensitive marker of renal function, especially in the early stages of diabetic nephropathy, and its sensitivity is due to serum creatinine [26, 27]. Alexandra-Mihaela Visinescu [28] et al. concluded that CKD is an important complication in diabetic patients, and cystatin C is more sensitive than serum creatinine in the assessment of renal function, and combining cystatin C with other biomarkers can improve the diagnostic accuracy of CKD in diabetic patients. Zhao Ping [29] et al. investigated the relationship between serum cystatin C level and renal microvascular perfusion in patients with DN, suggesting that CEUS parameter reflects the changes of renal microvascular perfusion in patients with DN, and AUCs may be a useful indicator of GFR decline in DN patients with elevated serum cystatin C.

As a crucial indicator of lipid metabolism, total cholesterol is closely related to the occurrence and development of diabetic nephropathy. Hypercholesterolemia can induce inflammation and oxidative stress, leading to the proliferation of mesangial cells and the damage of renal tubule interstitial [30]. Concurrently, kidney function damage can disrupt lipid metabolism, resulting in increased cholesterol levels. In addition, the increase of total cholesterol can induce atherosclerosis, leading to vascular complications such as renal artery lesions and consequently reducing renal blood perfusion. The interplay between these factors exacerbates the clinical manifestations of diabetic nephropathy, driving its deterioration [31].

In this study, a random forest model was constructed by combining contrast-enhanced ultrasound parameters and biochemical indexes with the method of machine learning. Xuee Su [32] et al. used a machine learning model containing two-dimensional ultrasound imaging and biochemical data to diagnose patients with type 2 diabetes mellitus (T2DM). By incorporating 10 ultrasonic features and biochemical indicators such as total cholesterol, triglyceride, blood creatinine and urea nitrogen, she constructed a model for prediction and evaluation, and obtained good sensitivity, specificity and accuracy of evaluation. In our study, all the patients included in this model had obtained renal pathological results, which served as the gold standard to guide the model assessment, providing a good orientation for the evaluation. And it has achieved good consistency and accuracy.

Limitations of this study: (1) The sample size was limited to retrospective studies from a single center; the predictive model developed in this paper may reflect the distribution of biomarkers in a specific population (such as serum creatinine, estimated glomerular filtration rate based on the EPI formula), but these distributions do not apply to cohorts with different genetic or environmental backgrounds; these findings require further multi-institutional validation with larger sample sizes; (2) this was a retrospective study that could not completely avoid missing data and measurement bias, so further studies must include more candidate biomarkers to develop predictive models in the future.

The diagnostic model developed in this study, which is based on eight key biomarkers, provides a practical tool for the differential diagnosis of diabetic nephropathy (DN) and non-diabetic renal disease (NDRD). Its core advantage lies in enabling noninvasive diagnosis using routine laboratory and examination indicators, effectively compensating for the limitations of renal biopsy. It is particularly suitable for patients with biopsy contraindications or those with poor physical conditions, and holds significant value in optimizing clinical workflows and alleviating the burden on patients. From the perspective of clinical translation, the model can be seamlessly integrated into existing diagnostic and treatment pathways by incorporating routine tests and embedding into clinical decision support systems (CDSS) [33], thereby playing a role in scenarios such as disease stratification and treatment monitoring.

Of course, future efforts could focus on incorporating multi-dimensional indicators to enhance its efficacy and optimizing through interpretable algorithms to improve clinical acceptance. Additionally, clinicians will receive training on interpreting the model’s output to improve diagnostic consistency, with emphasis on the model serving as a complement to, rather than a replacement for, clinical judgment. The interface will include visual aids to contextualize results, ensuring usability across diverse clinical settings.

In conclusion, our approach demonstrates the significant role of integrating machine learning with contrast-enhanced ultrasound and clinical data in model construction and the enhancement of disease detection strategies, which is poised to find widespread application across various medical disciplines.

## Data Availability

No datasets were generated or analysed during the current study.

## Abbreviations

AGEs: Advanced glycosylation end products
AI: Artificial intelligence
AUC: Area under the curve
CDSS: Clinical decision support systems
CEUS: Contrast-enhanced ultrasound
CIs: Confidence intervals
CKD: Chronic kidney disease
DN: Diabetic nephropathy
ICCs: Intraclass correlation coefficients
LOA: Limits of agreement
MTT: Mean transit time
NDRD: Non-diabetic renal disease
PE: Peak enhancement
PEAK: Peak intensity
ROC: Receiver operating characteristic
ROI: Region of interest
SD: Standard deviation
T2DM: Type 2 diabetes mellitus
TP: Time-to-peak
UACR: Urinary albumin to creatinine ratio
